# Effect of Heat-Inactivated *Clostridium sporogenes* and Its Conditioned Media on 3-Dimensional Colorectal Cancer Cell Models

**DOI:** 10.1038/srep15681

**Published:** 2015-10-28

**Authors:** Madhura Satish Bhave, Ammar Mansoor Hassanbhai, Padmaja Anand, Kathy Qian Luo, Swee Hin Teoh

**Affiliations:** 1 School of Chemical and Biomedical Engineering, Nanyang Technological University, Singapore

## Abstract

Traditional cancer treatments, such as chemotherapy and radiation therapy continue to have limited efficacy due to tumor hypoxia. While bacterial cancer therapy has the potential to overcome this problem, it comes with the risk of toxicity and infection. To circumvent these issues, this paper investigates the anti-tumor effects of non-viable bacterial derivatives of *Clostridium sporogenes*. These non-viable derivatives are heat-inactivated *C. sporogenes* bacteria (IB) and the secreted bacterial proteins in culture media, known as conditioned media (CM). In this project, the effects of IB and CM on CT26 and HCT116 colorectal cancer cells were examined on a 2-Dimensional (2D) and 3-Dimensional (3D) platform. IB significantly inhibited cell proliferation of CT26 to 6.3% of the control in 72 hours for the 2D monolayer culture. In the 3D spheroid culture, cell proliferation of HCT116 spheroids notably dropped to 26.2%. Similarly the CM also remarkably reduced the cell-proliferation of the CT26 cells to 2.4% and 20% in the 2D and 3D models, respectively. Interestingly the effect of boiled conditioned media (BCM) on the cells in the 3D model was less inhibitory than that of CM. Thus, the inhibitive effect of inactivated *C. sporogenes* and its conditioned media on colorectal cancer cells is established.

Colorectal carcinoma, characterized by the uncontrolled growth of cells in the epithelial tissue of the large intestine, is the third most common cancer in men, second most common cancer in women around the world and the second highest leading type of cancer deaths in the United States^1^. However, existing forms of cancer treatment are limited in their efficacy. Surgery is the first line of treatment for colorectal cancers detected in their early stage, but it is ineffective against the advanced stages of cancer^2^,^3^. The tumor microenvironment plays a key role in limiting the efficacy of other conventional forms of cancer treatment, such as chemotherapy and radiation therapy (RT). The necrotic (anoxic) core and hypoxic region are key features of the tumor microenvironment. As oxygen and the nutrient flow do not reach these parts of the tumor, their concentrations are much lower here than in normal tissues^4^,^5^. RT involves the use of ionizing radiation to curb the growth of cancer cells by forming free-radical debris of DNA. Oxygen molecules react with the free-radical DNA debris to make the DNA damage permanent and bring about cell death. This makes the efficacy of RT heavily dependent on the presence of oxygen and thus, intra-tumoral hypoxia greatly curbs the effectiveness of RT in treating tumors^6^. Hypoxia also compromises on the efficacy of chemotherapy. There are various reasons for this. Firstly, these hypoxic tumor regions are located far away from the blood vessels, preventing the delivery of chemotherapeutic drugs to cells^7^,^8^. Secondly, some drugs such as melphalan^9^,^10^, bleomycin^11^ and etoposide^12^,^13^ require cellular oxygen to bring about cell death and are therefore ineffective in hypoxic conditions. Finally, alkylating agents and anti-metabolite anti-cancer drugs only act against rapidly proliferating cells and because hypoxia slows down the cell-cycle, these drugs cannot effectively cause cancer cell death either^7^,^14^. The limitations of existing cancer treatment methods have led to a pressing need to explore alternative treatment methods that will overcome the hypoxic barrier of tumors and be effective in targeting cancer.

Bacterial cancer therapy has the potential to overcome these limitations and provide a viable alternative to existing treatment modalities^15^. The hypoxic conditions of the tumor microenvironment, that are a huge obstacle for RT and chemotherapy, were recognized as a potent tool for bacterial cancer therapy. This is because such conditions are perfect for the growth of anaerobic bacteria, which accumulate and proliferate in the hypoxic regions of the tumor before their natural cytotoxicity induces cancer cell death^16^,^17^. Clostridial strains have been at the center of bacterial tumor therapy since the 19^th^ century because of the ability of their spores to selectively germinate in the hypoxic cores of tumors^18^,^19^. *Clostridium sporogenes,* a proteolytic species, is reported to have a superior ability of tumor colonization^20^,^21^. Wild-type clostridial spores have been found to exert oncolytic effects on tumors^22^,^23^, clostridial spores combined with other cancer therapies were found to have an enhanced anti-cancer effect^24^,^25^, and genetically modified clostridial species have also been used in *Clostridium*-directed enzyme prodrug therapy (CDEPT)^16^,^20^,^21^,^26^ . Secreted bacterial products that are found in the conditioned media (CM) of bacterial cultures have been investigated and several studies have shown that they also have an anti-tumor effect^27^,^28^. Amongst clostridial products, *Clostridium perfringens* enterotoxin (CPE) has been studied extensively and found to interact with claudin-3 and -4 receptors that are overexpressed in many types of tumors, to trigger cancer cell death^29^,^30^. Despite these advances, clostridial cancer therapy has not gained widespread acceptance as a potential treatment method.

This is because clostridial cancer therapy has limitations of its own. The administration of this spore-forming pathogenic bacteria poses a high risk of infections and toxicity to patients after the spores have germinated. Several studies involving the administration of clostridial spores to tumor-bearing animals resulted in their death due to bacterial infections or the toxicity of the bacteria^16^,^31^,^32^. In two recent studies *C. novyi-NT* spore treatment, one involving canines and the other involving canines and a human patient with a leiomysarcoma, the administered dose of the spores caused a severe case of toxicosis^33^,^34^. Additionally, using anaerobic bacteria also leaves a viable rim of tumor cells which increases the chance of tumor recurrence^22^,^35^,^36^,^37^. Another point to note is that *in vitro* studies done in the field of bacterial cancer therapy are performed using 2-dimensional (2D) cancer cell cultures^38^,^39^. This leads to a potential issue of reliability as these 2D cultures are not representative of *in vivo* tumor conditions, thus making the findings of such studies less physiologically relevant^40^,^41^,^42^.

There is a need to develop a form of bacterial cancer therapy that minimizes the risk of infection and is tested on a physiologically relevant *in vitro* platform, before advancing on to *in vivo* studies. In an attempt to overcome these gaps, non-viable derivatives of bacteria are used, which pose a much lower risk of infection as compared to spores or live bacteria. Past studies have shown that bacteria can retain their natural cytotoxicity even after they have been heat-inactivated^38^,^43^. This paper studies the effect of heat-inactivated *C. sporogenes* bacteria (IB) on colorectal cancer cells for the first time, using CT26 and HCT116 cell lines. Additionally, the effect of the secreted bacterial products of *C. sporogenes* in conditioned media is investigated. The inhibitive effect of IB and CM was tested on CT26 and HCT116 cells cultured in a 3-dimensional (3D) spheroid model that simulates the tumor microenvironment. We hypothesize that the non-viable derivatives of *C. sporogenes*, IB and CM, can inhibit colorectal cancer cells.

## Results

### Heat-Inactivation kills bacterial cells

**Figure 1:**
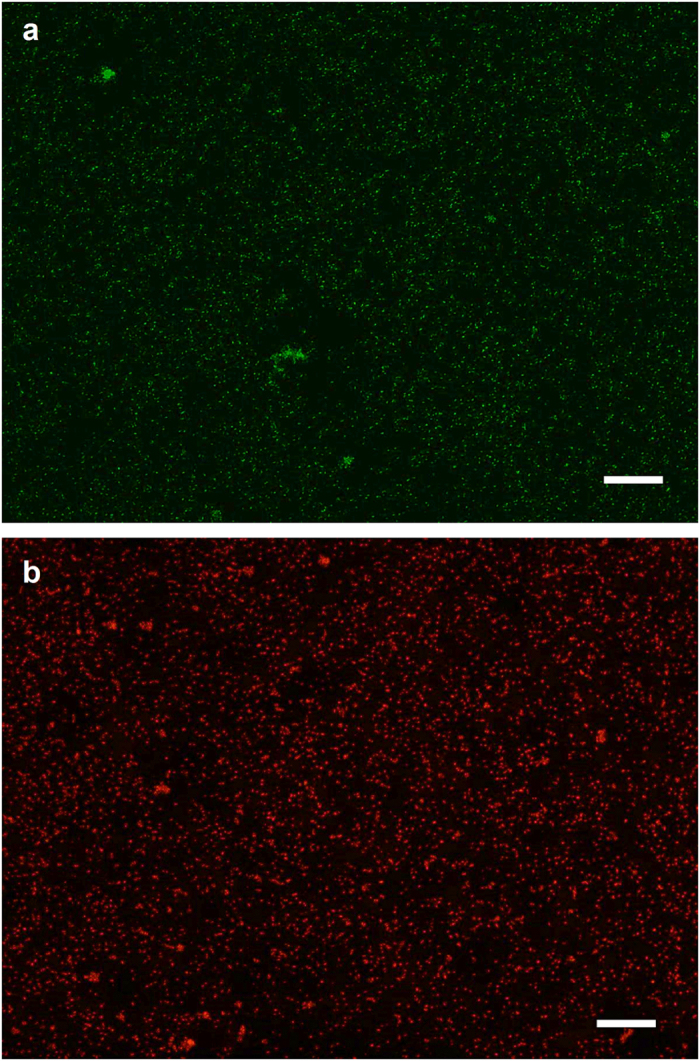
Fluorescence image of cell viability of *C. sporogenes* after heat inactivation. DMAO stains bacterial cells green and EthD-III stains the non-viable bacterial cells red. (**a**) Bacteria before heat-treatment. (**b**) Bacterial cells after 80 °C heat inactivation for 2 hours. Scale bar represents 100 μm.

*C. sporogenes* were heat-inactivated to minimize the risk of infection. Live bacteria and heat-inactivated bacteria were tested with the bacterial viability assay, using DMAO and Ethidium homodimer-III (EthD-III) dyes. DMAO can permeate cell membranes of both live and dead bacteria, but EthD-III can only permeate dead bacterial cells. As shown in Fig. 1a, live vegetative bacteria was stained green with DMAO. After heat-inactivation, the bacteria in the sample got stained red with EthD-III, indicating that the heat treatment is effective (Fig. 1b).

### Inactivated *C. sporogenes* and its conditioned media inhibit colorectal cancer cells in 2D culture

**Figure 2:**
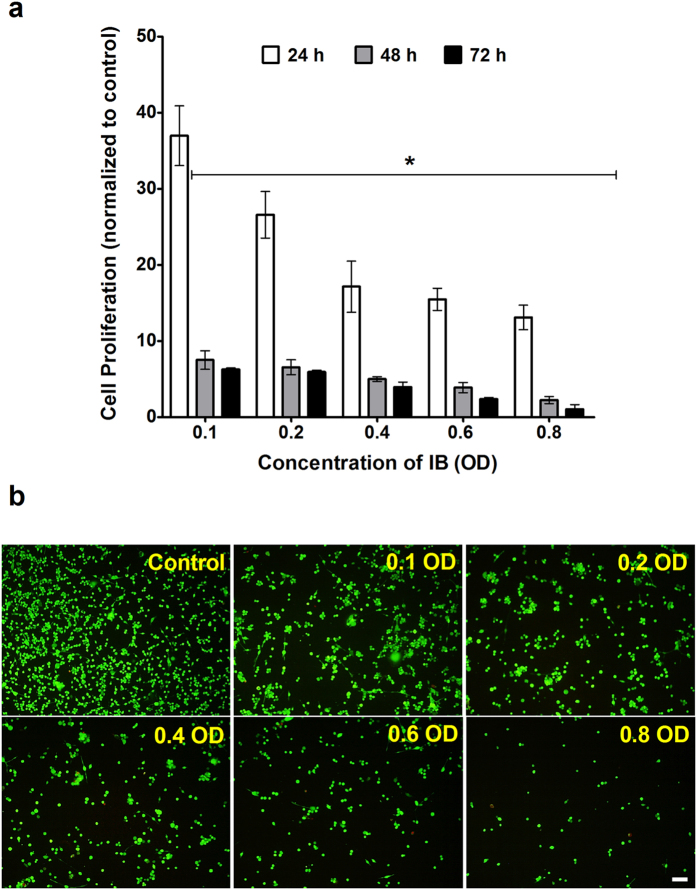
Effect of IB on 2D cell culture. (**a**) Cell proliferation with WST-1 assay of 2D culture of CT26 cells exposed to varying concentrations of inactivated bacteria. 0.1 OD = 2.4 × 10^6^ bacterial cells/ml. *t*-test is a comparison with control. (*p < 0.005) (**b**) Fluorescence microscopy with Calcein AM and Ethidium homodimer I (EthD-I), of cell viability of CT26 cells after they had been exposed to inactivated bacteria at varying concentrations for 24 hours. Image of cells in top left quadrant of each well. Scale bar represents 200 μm.

The 2D monolayer cultures of CT26 and HCT116 cells were exposed to varying concentrations of IB and the cell proliferation rate was expressed as a percentage of the control. At all the concentrations of IB, there was a significant decrease in the cell proliferation rates. It is important to note that even the lowest concentration of IB (0.1 OD) inhibits the cell proliferation of the CT26 cells to 37.0% of the control after just 24 hours. At the 48 and 72 hour time-point, the cell proliferation of the sample is reduced to 7.5% and 6.3% respectively (Fig. 2a). On the other hand, when exposed to an eight times higher concentration of IB (0.8 OD), the proliferation of cells decreased significantly to 13.1%, 3.0% and 2.0% at 24, 48 and 72 hours respectively. Correspondingly, the viability of the cells decreased in a dose dependent manner. This is validated by the fluorescence images demonstrating that an increase in IB concentrations leads to decrease in cell viability (Fig. 2b). Supplementary Fig. S1 shows that 0.1 OD of IB reduces the cell proliferation of HCT116 cells to 97.0%, 77.0% and 44.3% at 24, 48 and 72 hours respectively. Additionally, 0.8 OD of IB reduces HCT116 cell proliferation to 40.4%, 1.49% and 0.63% at 24, 48 and 72 hours respectively.

**Figure 3:**
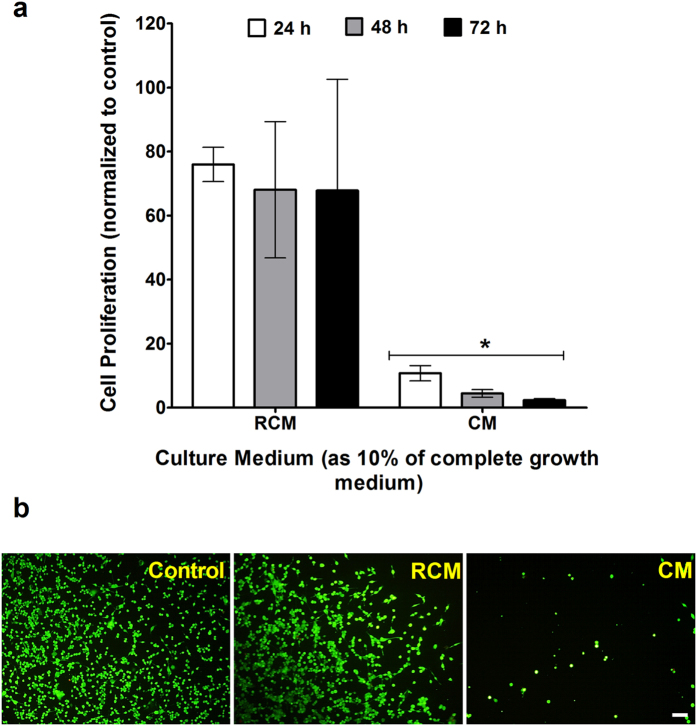
Effect of CM on 2D cell culture. (**a**) Cell proliferation with WST-1 assay of 2D culture of CT26 exposed to 10% of Conditioned Media (CM) of *C. sporogenes* and Reinforced Clostridium Media (RCM). *t*-test is a comparison with control. (*p < 0.005) (**b**) Fluorescence assay of cell viability of CT26 cells after they had been exposed to 10% of RCM and CM for 24 hours. Image of cells in top left quadrant of each well. Scale bar represents 200 μm.

After establishing the effect of IB on cells, the bacteria-free CM was studied in comparison to RCM, which is the un-inoculated bacterial media (Fig. 3). Cell proliferation of CT26 cells exposed to 10% CM decreased to 10.8% of the control in the first 24 hours, after which it further decreased to 4.5% (48 hours) and 2.4% (72 hours). 10% CM inhibits the cell proliferation of HCT116 to 55.9%, 30.6% and 7.4% at 24, 48 and 72 hours respectively (Supplementary Fig. S1). In comparison, the cell proliferation rate of CT26 cells exposed to 10% RCM is 76.0% of the control at the first time point and it decreases to 68.1% (48 hours) and 67.9% (72 hours) later (Fig. 3a) and that of HCT116 is 83.2% at 24 hours, 72.8% at 48 hours and 75.4% at 72 hours (Supplementary Fig. S1). The fluorescence images of the cells, support the cell proliferation results (Fig. 3b). They show that the amount of viable cells remaining after exposure to CM is much lower than those exposed to RCM.

### Inactivated *C. sporogenes* and its conditioned media inhibit colorectal cancer cells in 3D culture

**Figure 4:**
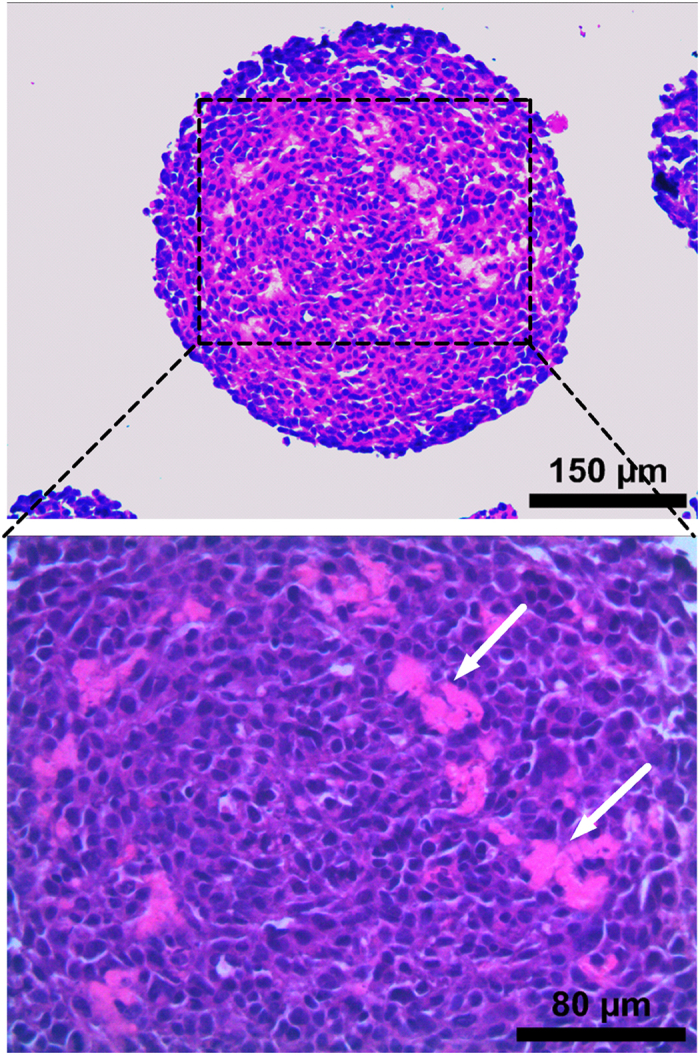
H&E staining of 3D spheroids of CT26, at 72 hours. Arrows indicate necrotic regions formed within spheroid, stained only with eosin.

After having examined these non-viable derivatives on a 2D monolayer culture, its effects on a 3D spheroid culture were studied. H&E staining was conducted on CT26 spheroids grown for 72 hours and an absence of haematoxylin was observed in some regions of the spheroids. This indicates a lack of nuclei in the region, suggesting that the cells in this region were necrotic (Fig. 4).

**Figure 5:**
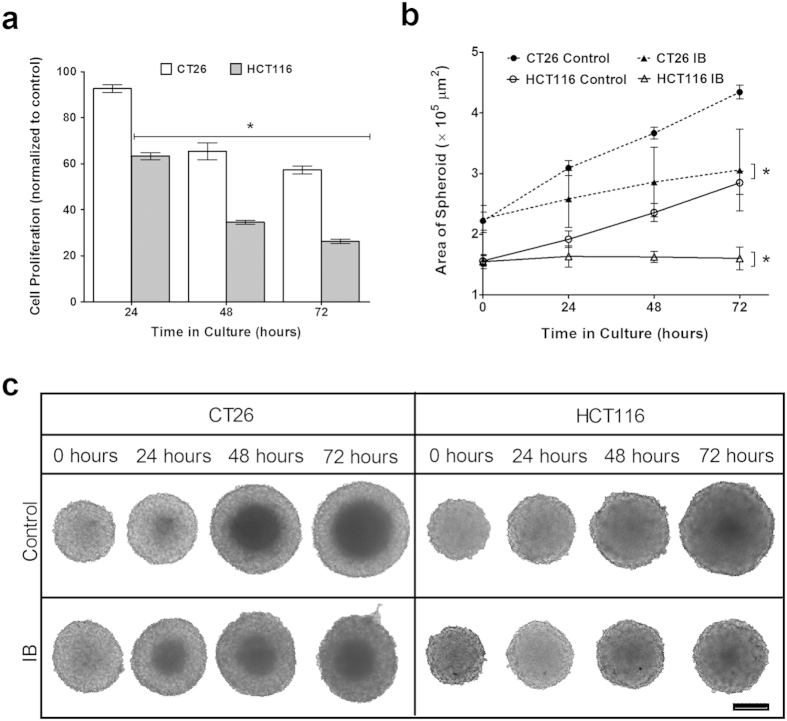
Effect of IB on 3D spheroids. (**a**) Cell proliferation with WST-1 assay of CT26 and HCT116 spheroids exposed to 0.1 OD concentration of inactivated bacteria over 72 hours. 0.1 OD = 2.4 × 10^6^ bacterial cells/ml. *t*-test is a comparison with control. (*p < 0.005) (**b**) Area of 3D spheroids after incubation with 0.1 OD of inactivated bacteria, at the 0, 24, 48 and 72 hour time points. The area (μm^2^) of the spheroids in the images was measured using ImageJ software. *t*-test is a comparison with control (*p < 0.005, at all time points). (**c**) Effect of 0.1 OD of inactivated *C. sporogenes* on 3D spheroids, compared with Control. Scale bar represents 1000 μm.

The spheroids were exposed to IB (0.1 OD) over a period of 72 hours. The cell proliferation was measured using the WST-1 assay at 24 hour intervals (Fig. 5a). At each time point, a decrease in the cell proliferation was observed. At 72 hours, the cell proliferation of CT26 spheroids reduces to 57.3% and that of HCT116 reduces to 26.2%. These values are higher than the cell proliferation of cells in the 2D model exposed to the same concentration of IB. As anticipated, the size of the control spheroids increases significantly between each time point, over the entire duration of the study. 0.1 OD IB appears to stunt the spheroid growth, with the increase in the area of the spheroids being marginal at each time point (Fig. 5b). Over time, there is a significant difference in the area of the spheroids and at the end of 72 hours, the CT26 spheroids exposed to IB (3.25 × 10^5^ μm^2^) are 25% smaller than the control spheroids (4.34 × 10^5^ μm^2^) and the HCT116 spheroids exposed to IB (1.60 × 10^5^ μm^2^) are 44% smaller than the control (2.85 × 10^5^ μm^2^). The inhibitory effect of higher concentrations of IB (0.4 OD and 0.8 OD) on CT26 and HCT116 spheroids, shown in Supplementary Fig. S2, is greater than that of 0.1 OD IB. 0.4 OD IB causes a 32% decrease in CT26 spheroid area and a 50% decrease in HCT116 spheroid area. 0.8 OD IB causes a 33% and 60% decrease in CT26 and HCT116 spheroid area respectively. Moreover, it is interesting to note that despite the increase in size, the shape of the control spheroids is maintained throughout the study, while the shape of the CT26 spheroids exposed to IB starts to get deformed after the 48 hour time point (Fig. 5c).

**Figure 6:**
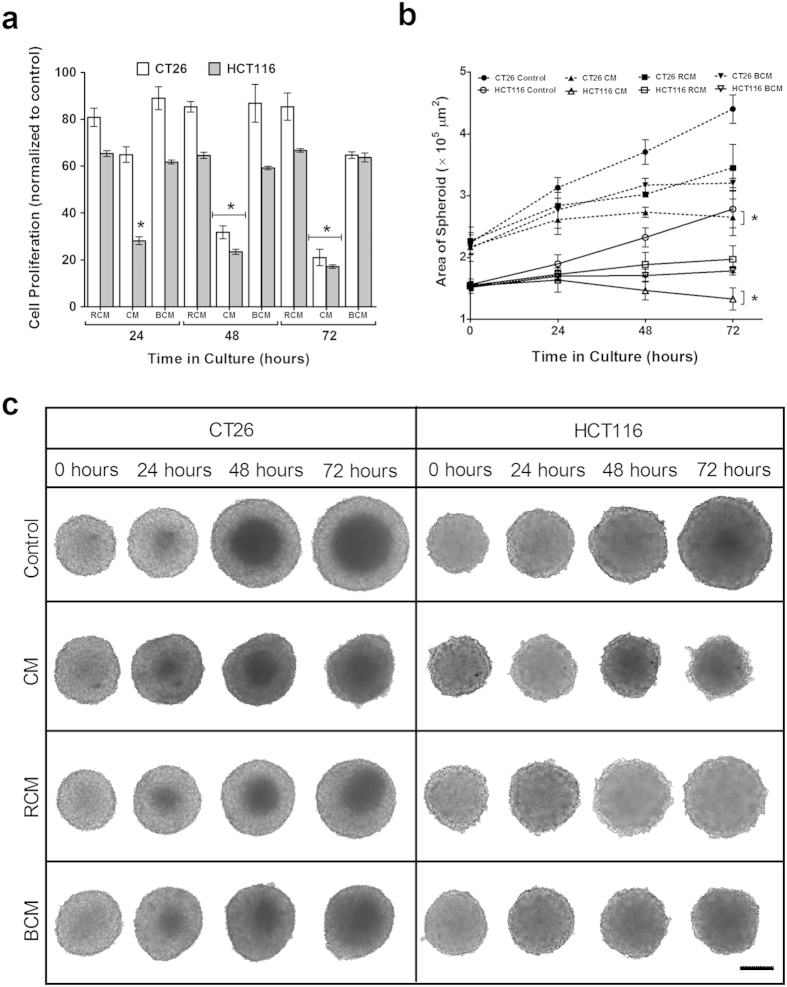
Effect of CM on 3D spheroids. (**a**) Cell proliferation with WST-1 assay of CT26 and HCT116 spheroids exposed to 10% of Conditioned Media (CM) of *C. sporogenes* and Reinforced Clostridium Media (RCM). *t*-test is a comparison with control. (*p < 0.005). (**b**) Area of spheroids after incubation with 10% RCM, CM and BCM of *C. sporogenes* at the 0, 24, 48 and 72 hour time points. The area (μm^2^) of the spheroids in the images was measured using ImageJ. *t*-test is a comparison with control (*p < 0.005, at all time points). (**c**) Effect of 10% CM of *C. sporogenes* on 3D spheroids, compared with Control, 10% RCM and 10% BCM. Scale bar represents 1000 μm.

The effect of CM on the spheroids was investigated similarly. RCM caused a slight decrease in the cell proliferation across all time points. On the other hand, the inhibitive effect of CM is significant, with the cell proliferation of CT26 spheroids decreasing to 20.0% and that of HCT116 spheroids decreasing to 17.0% after 72 hours (Fig. 6a). At the end of the study, the area of the CM exposed CT26 spheroids is 38% smaller than the area of the control spheroids, while the area of CM exposed HCT116 spheroids is 53% smaller than the control spheroids (Fig. 6b). CM induces a slight regression in the CT26 spheroids from 2.73 × 10^5^ μm^2^ at 48 hours to 2.67 × 10^5^ μm^2^ at 72 hours. Additionally, CM causes an overall regression of the HCT116 spheroids from 1.54 × 10^5^ μm^2^ at 0 hours to 1.33 × 10^5^ μm^2^ at 72 hours. For RCM exposed spheroids, the area, although smaller than the control, was still larger than the spheroids exposed to CM. This inhibitive effect of CM is also reflected the morphology of the spheroids (Fig. 6c). CM causes deformation of the spheroids, stunting of the spheroid size initially and then its regression. In contrast, RCM maintained overall spheroid shape as compared to CM exposed spheroids.

### Heat-sensitive proteins in *C. sporogenes* Conditioned Media responsible for inhibiting CT26 cells

In an attempt to determine if proteins are involved in the previous observations, CM was boiled to denature the proteins and obtain boiled conditioned media (BCM). 3D spheroids were exposed to BCM in a 72 hour study. By the end of the study, it was observed that BCM has a considerably lesser inhibitive effect on cell proliferation (64.7% for CT26 spheroids, and 63.7% for HCT116 spheroids) than CM (20.0% for CT26 spheroids and 17.0% for HCT116 spheroids) (Fig. 6a). The area of the CT26 and HCT116 spheroids exposed to BCM is consistently higher than that of the spheroids exposed to CM. Furthermore, the area of BCM exposed spheroids is similar to that of the area of spheroids exposed to RCM (Fig. 6b). The growth of the spheroids exposed to BCM is stunted, although their shape is maintained. Comparatively, spheroids exposed to CM regress in size and also lose their shape (Fig. 6c).

### Inactivated *C. sporogenes* break down the ECM in 3D spheroids

**Figure 7:**
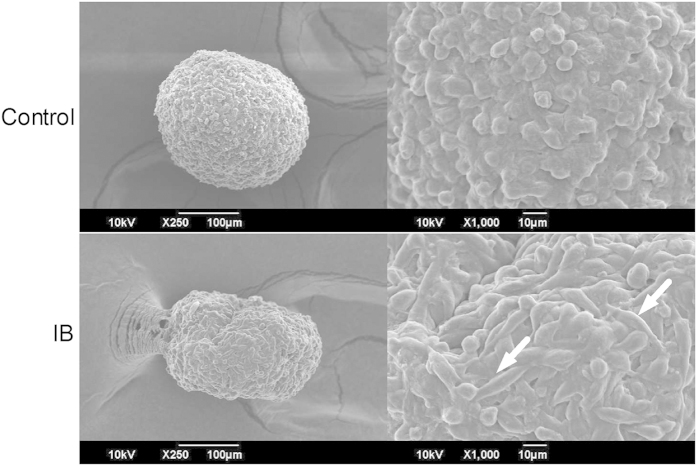
Comparison between morphology of CT26 Control spheroid and spheroid exposed to IB after 24 hours. Images taken with scanning electron microscope (SEM). Arrows indicate elongated cells on the surface and the white arrow indicate deformation in the shape of IB-exposed spheroids.

To further characterize the role of IB in inhibiting cancer cell growth, the morphology of the 3D spheroids was examined. It was found that the cells on the periphery of the control CT26 spheroids were circular in shape, packed together with tight cell contacts and thickly covered by the ECM. On the other hand, on the periphery of the spheroids exposed to IB, the cells were elongated in shape, loosely packed and the ECM appeared to be thinner. The spherical morphology of the control spheroid was not observed in the IB spheroid, which appeared deformed (Fig. 7). This is in agreement with Fig. 5c, where the IB-exposed CT26 spheroids appeared to have lost is morphological integrity at 72 hours.

## Discussion

There exist several studies on the oncolytic effect of *C. sporogenes* spores, the earliest of which was conducted in 1935 by Connell *et al.* Since then, *C. sporogenes* spores have been genetically modified for use in pro-drug therapy or the delivery of therapeutic agents^16^,^20^,^21^,^26^,^44^,^45^. However, the risk of infection persisted with the application of spores or live vegetative bacteria. Therefore, this study investigated the anti-cancer effect non-viable derivatives of *C. sporogenes*, as they do not possess the ability to cause infections in the same way as spores or live, vegetative cells. Our findings suggest that the observed inhibitive effect on the cells can be attributed to the natural oncolytic properties of IB and CM as these bacterial derivatives cannot proliferate and do not deprive the cells of nutrition.

While the inactivated forms of gram-negative *Streptococcus equisimilis* and *Staphylococcus aureus* bacteria have been studied previously by Klier *et al.* in 2011^43^, this paper examines the effect of heat-inactivated *C. sporogenes* on colorectal cancer cells for the first time. A range of temperatures was initially investigated (data not shown) but 80 °C was the most ideal as the bacteria were not only inactivated, but more importantly still intact. This is crucial as we can then associate the effects observed to the bacterial surface proteins instead of the intracellular components. To study the effects of CM, a range of RCM concentrations, diluted in cell culture media, was examined to determine the maximum concentration that can be used without adversely affecting the cell viability. Mammalian cells cannot grow in bacterial culture media and as expected, the RCM components affected cell viability to some extent. 10% RCM in cell culture media was found to be the most ideal and thus used to compare against 10% CM in cell culture media for subsequent experiments.

In the 2D model, the exposure to IB exerted a significant anti-proliferative effect on CT26 and HCT116 cells in a concentration-dependent manner and CM was found to also have a strong inhibitive effect on the cells. CT26 cells were found to be more sensitive to IB and CM in the 2D model. In a study by Arimochi *et al.* 10% CM of *C. perfringens* was administered to colorectal cancer cells, which resulted in a 60% decrease in cell proliferation after 4 days^38^. Together with our data, this demonstrates the potential of the bacteria-free CM as an anti-cancer agent.

Despite these promising results, it has been suggested in literature that cells grown in a 2D monolayer are more susceptible to drug treatment and thus not a reliable *in vitro* model^40^,^42^. The use of 3D spheroids in this study is important as it allows the evaluation of IB and CM as potential anti-cancer agents on a physiologically relevant platform^46^,^47^. Methods such as hanging-drop^48^ and low adhesion plates^49^ were tried initially to generate the 3D spheroids, but these methods were found to have limitations which affected the uniformity of the spheroids. Later, the use of round-bottom, low-adhesion plates generated spheroids that were consistently reproduced with uniformity in both size and shape. The spheroids that were grown developed necrotic regions that simulate the hypoxic, necrotic conditions of the *in vivo* tumor microenvironment^47^,^50^. A 3D model is more complex as it simulates the cell-cell and cell-ECM interactions of real tumors^46^.

This could be a reason why the cell proliferation rate of the CT26 spheroids exposed to 0.1 OD of IB is approximately 9 folds higher than that of its 2D model. In general, the cells grown in a monolayer in a 2D model are more susceptible to external treatments as compared to that of a spheroid where there is a gradient of nutrient and gas exchange with the surroundings^41^,^42^. SEM imaging was used to understand the morphological changes caused by IB in the spheroid. It was found that the spheroid morphology was lost and the cells were loosely packed. This suggests that IB inhibits the proliferation of cells by breaking down the ECM and destroying the cell-ECM interactions. Furthermore, *in vivo* studies have demonstrated that inactivated bacteria of *S. aureus* have been able to elicit an immune response that led to a delay in tumor growth when applied both locally and systemically^43^.

The effect of CM on CT26 and HCT116 cell was highly inhibitive while the presence of RCM was shown to have had a lesser inhibitive effect on the cells and spheroids. Therefore, the main factor in affecting anti-proliferation of the cells was likely to be a bacterial product secreted into the culture medium. This was confirmed by comparing the effect of CM with that of BCM. BCM’s inhibitive effect was diminished and restored to levels similar to that of RCM. This suggests that the bacterial proteins in CM have caused this inhibitive effect. In particular, this could be due to the extracellular proteases of *C. sporogenes,* one of which is collagenase, that degrade the tumor tissue^51^,^52^.

This study has discovered that both these non-viable bacterial derivatives of *C. sporogenes* have very promising inhibitive effects on colorectal cancer. However, the mechanism of inhibition and the interaction between IB and cancer cells still remains to be understood. It is interesting to note that HCT116 spheroids are more sensitive to IB and CM than the CT26 spheroids. Understanding the differences between the two cell lines may help to better characterize the mechanism of inhibition. Other studies have investigated CM and IB in different bacteria species but have not been able to elucidate the mechanism of the observed actions^38^,^43^. Earlier studies have shown that the use of bacterial components may be applied as an adjuvant to initiate anti-tumor responses^53^. Additionally, the specific components of CM that causes cancer cell inhibition has also not been identified although the data suggests that it is heat-liable in nature. It will be important to address these unanswered questions before these bacterial derivatives can be developed into effective therapeutic agents. In conclusion, this study proves that inactivated *C. sporogenes* and its conditioned media are both non-viable bacterial derivatives that can inhibit colorectal cancer cells and carry the potential for therapeutic use.

## Materials and Methods

### 2D Cancer Cell Culture

The CT26 murine colorectal cancer cells (ATCC CRL-2638) and HCT116 human colorectal cancer cells (ATCC CCL-247) were purchased from the American Type Culture Collection. The complete growth medium used for the CT26 cell culture was the Roswell Park Memorial Institute (RPMI) culture medium (Hyclone, USA) and that for the HCT116 cell culture was McCoy’s 5A medium (Sigma-Aldrich, USA), supplemented with 10% Foetal Bovine Serum (FBS) (Gibco, USA) and 1% Penicillin-streptomycin (Gibco, USA). The cells were cultured in flasks (Corning, USA) in their respective complete growth media, incubated in a humidified atmosphere at 37 °C, 5% CO_2_. They were grown to 70–80% confluence before being passaged. The cells were seeded in 96-well tissue culture plates at a density of 5000 cells/well, and were cultured overnight to allow them to adhere to the bottom of the wells in monolayers before being used for the experiments.

### 3D Spheroid Culture

The CT26 and HCT116 cells were cultured in round-bottomed, ultra-low adhesion 96-well plates (Corning, USA). The complete growth medium used for the 3D spheroid culture was the RPMI culture medium (Hyclone, USA) for CT26 cells and McCoy’s 5A culture medium (Sigma-Aldrich, USA) for HCT116 cells, supplemented with 10% (FBS) (Gibco, USA) and 1% Penicillin-streptomycin (Gibco, USA). 200 μl of the complete growth medium was added to each well and 1500 cells were seeded in each well. The cells were spun at 986 g for 10 minutes using a plate centrifuge (Thermo Scientific) to facilitate their collection at the bottom of the wells. The 3D spheroid culture was incubated at 37 °C, 5% CO_2_ for a period of 3 days to allow the development of stable spheroids with necrotic regions that simulate *in vivo* tumors. After this 3 day growth period, the spheroids were used for the experiments.

### Bacterial Culture and Inactivation

The *C. sporogenes* spores (ATCC 13732) were purchased from the American Type Culture Collection. 15 μl of the spore suspension was added to 15 ml of Reinforced Clostridial Media (RCM) (Oxoid, England), in petri dishes. An anaerobic chamber was set-up following the protocol of the GasPak™ EZ Anaerobic Container System (Becton, Dickinson and Company, USA). The bacterial culture was placed in the anaerobic chamber and incubated at 37 °C, 5% CO_2_ for 3 days. The bacterial culture was then harvested from the anaerobic chamber and heat-inactivated by placing it in an 80 °C water bath for 2 hours. After heat-inactivation, the optical density of the bacterial suspension was measured using UV-vis spectrophotometer (VWR, USA) quantify the amount of bacteria present in a 1 ml sample of the culture at 600 nm^43^. The culture was centrifuged at 2500 g for 20 minutes^28^ to pellet the bacteria. After the supernatant was discarded, the inactivated bacterial pellet was re-suspended in complete growth media of cancer cells for use in the experiments.

### Bacterial Viability Assay

The heat-inactivated *C. sporogenes* bacteria were subjected to a bacterial viability test to confirm the effectiveness of the heat-inactivation process. The Viability/Cytotoxicity Assay kit for Bacterial Live & Dead Cells (Biotium, USA), was used to stain the IB using fluorescent nucleic acid dyes following the manufacturer’s protocol. DMAO is a green dye that stains both live and dead bacteria while Ethidium Homodimer-III (EthD-III) is a red dye that only stains dead bacteria. Images were obtained using a fluorescence microscope (Olympus, Japan).

### Preparation of Conditioned Media and Boiled Conditioned Media

The spores were cultured in RCM using the same protocol as described above. After 3 days, the bacterial culture was removed from the anaerobic chamber and centrifuged at 2500 g for 20 minutes^28^ to pellet the bacteria. The supernatant was collected and filtered using a 0.20 μm filter. The result is defined as conditioned media (CM). The CM was placed in a water bath at 100 °C for 30 minutes to obtain boiled conditioned media (BCM).

### Cell Proliferation Assay

The inactivated bacteria (IB) suspension in complete growth medium was serially diluted to concentrations of 0.1, 0.2, 0.3, 0.4, 0.6 and 0.8 OD (measured at 600 nm). The CM sample was prepared as 10% of CM in complete growth media (10% CM). For the 2D cell culture, cells were grown in 96 well-plates overnight at a seeding density of 5000 cells/well. They were then incubated at 37 °C, 5% CO_2_ with 200 μl of the 10% CM test samples and IB test samples of varying concentration over a 72 hour period. For the 3D spheroid culture, 3 day old spheroids were incubated with 0.1 OD IB, 10% CM and 10% BCM test samples over a 72 hour period. In both 2D and 3D experiments, two controls was maintained where cells or spheroids were incubated with 200 μl of complete growth media and with 200 μl of 10% RCM. At each 24 hour time point, the test samples in each well were replaced with fresh RPMI media. The 3D spheroids were re-suspended into a single cell suspension. Cell Proliferation Reagent WST-1 (Roche, USA), was added to each well in a 1:10 ratio and the cells were incubated for 2 hours under culture conditions. The absorbance of the samples was measured using a microplate reader (BioRad, USA) at 430 nm with a reference wavelength of 650 nm. The absorbance value of each well is directly proportional to the number of viable cells.

### Cell Viability Assay

Cells, grown overnight in 96 well-plates at a seeding density of 5000 cells/well, were incubated at 37 °C, 5% CO_2_ with IB test samples at varying concentrations of 0.1, 0.2, 0.3, 0.4, 0.6, 0.8 OD and 10% CM over a 72 hour period. LIVE/DEAD® Viability/Cytotoxicity Kit for mammalian cells (Invitrogen, USA), containing Calcein AM and Ethidium homodimer –I (EthD-I) was used to determine the cell viability assay of the 2D cell culture. At each 24 hour time point, the test samples were removed and the wells were washed with PBS. 100 μl of the fluorescence dye working solution was added to each well and the microplate was incubated in darkness at room temperature for 30 minutes. A fluorescence microscope (Olympus, Japan) was used to take the fluorescence images of each well.

### Spheroid Area Measurement

3 day old spheroids were incubated at 37 °C, 5% CO_2_ with 0.1 OD IB, 10% CM and 10% BCM over a 72 hour period. At each 24 hour time point, the spheroids were imaged using a bright-field microscope (Olympus, Japan) and the diameter was measured using the Image J software.

### Histology

3D spheroids were initially grown for 3 days and were incubated in the presence/absence of IB for an additional 3 days. The spheroids were then harvested and fixed in 10% paraformaldehyde (PFA). They were then dehydrated with a series of increasing concentrations of ethanol before being embedded in paraffin. The embedded samples were sectioned to a thickness of 5.0 μm and mounted onto polysine slides. The sections were then stained with haematoxylin and eosin and imaged using a bright-field microscope.

### SEM Imaging

3 day old spheroids were grown in complete growth media and 0.1 OD of IB for another 72 hours were fixed in PFA and dehydrated with a series of increasing ethanol concentrations. Once the samples were completely dehydrated, they were coated with platinum for 60 seconds with a 20 mA coating current and imaged with a Scanning Electron Microscope (JEOL, Japan).

### Statistical Analysis and Data Processing

All experiments were performed in triplicate, for statistical significance. All data were expressed as Mean ± Standard Deviation of three separate experiments. Statistical analysis was conducted using two-tailed Student’s *t*-test.

## Additional Information

**How to cite this article**: Bhave, M. S. *et al.* Effect of Heat-Inactivated *Clostridium sporogenes* and Its Conditioned Media on 3-Dimensional Colorectal Cancer Cell Models. *Sci. Rep.* **5**, 15681; doi: 10.1038/srep15681 (2015).

## Supplementary Material

Supplementary Information
