# Supplementary material for: Effect of Heat-Inactivated Clostridium sporogenes and Its Conditioned Media on 3-Dimensional Colorectal Cancer Cell Models
Source: Sci Rep. 2015 Oct 28;5:15681. doi: 10.1038/srep15681 (PMC4623472; doi:10.1038/srep15681)
Supplement: Supplementary Information [file srep15681-s1.pdf]

Supplementary Information

Effect of Heat-Inactivated *Clostridium sporogenes* and Its Conditioned Media on 3-Dimensional Colorectal Cancer Cell Models.

Madhura Satish Bhave, Ammar Hassanbhai, Padmaja Anand, Kathy Qian Luo, Teoh Swee Hin*

School of Chemical and Biomedical Engineering, Nanyang Technological University, Singapore.

Correspondence and requests for materials should be addressed to T.S.H. (email: teohsh@ntu.edu.sg)

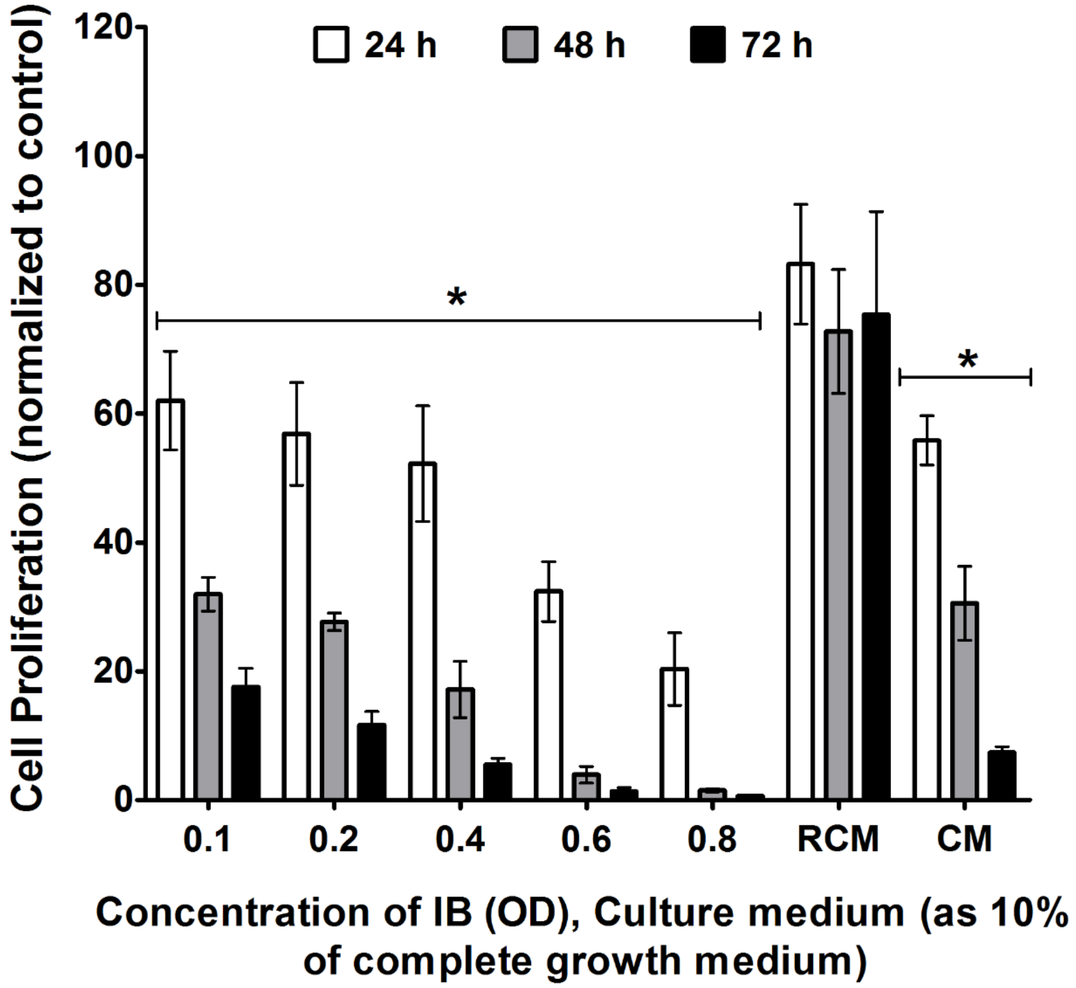

Supplementary Figure S1. **Effect of IB and CM on 2D culture of HCT116 cells**. Cell proliferation with WST-1 assay of 2D cell culture exposed to varying concentrations of inactivated bacteria, 10% of Conditioned Media (CM) of *C. sporogenes* and 10% Reinforced Clostridium Media (RCM). 0.1 OD = 2.4×10^6 bacterial cells/ml. *t*-test is a comparison with control. (* $p< 0.005$)

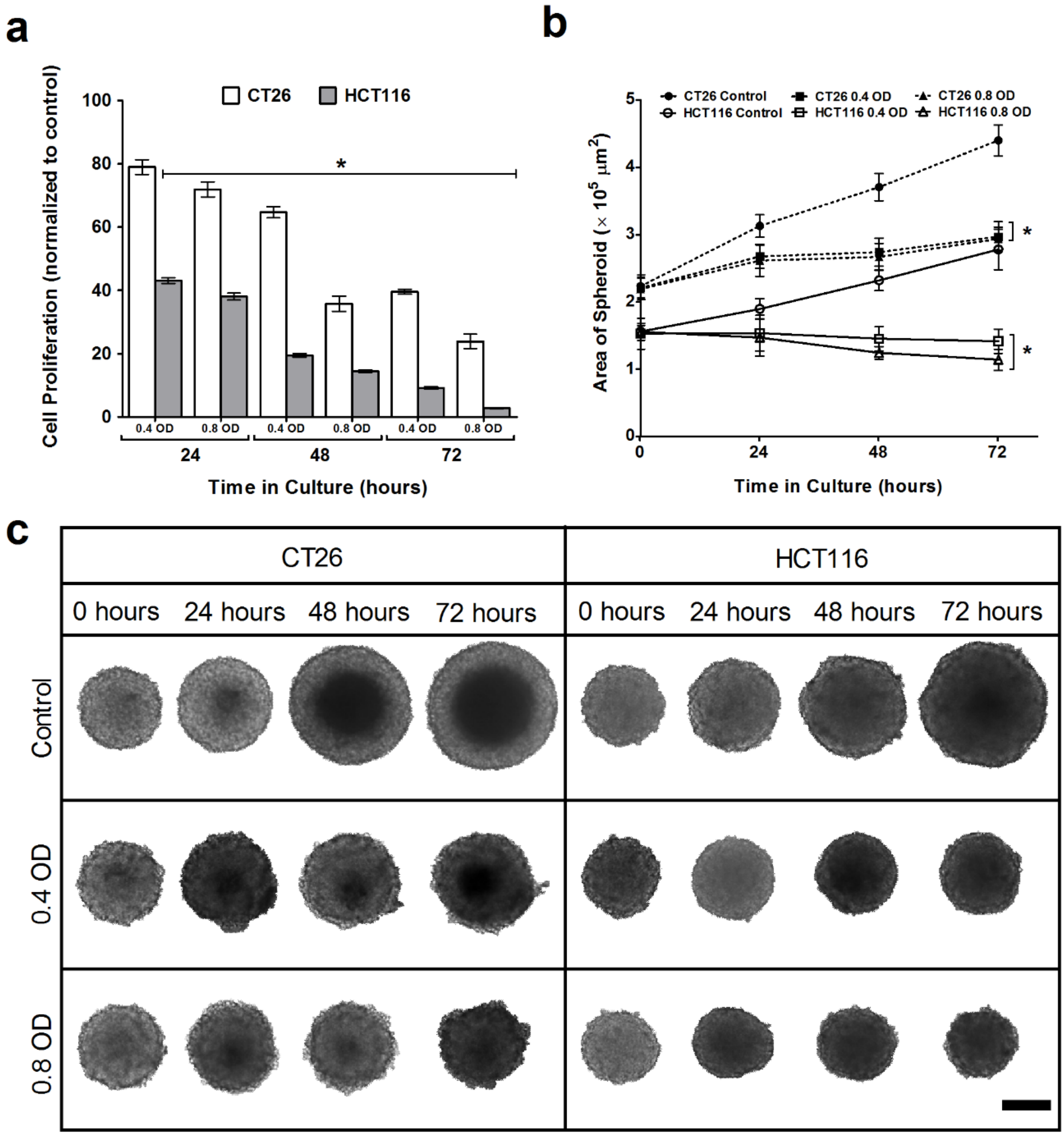

Supplementary Figure S2. **Effect of 0.4 OD and 0.8 OD IB on 3D spheroids.** (a) Cell proliferation with WST-1 assay of CT26 and HCT116 spheroids exposed to 0.4 OD and 0.8 OD concentration of inactivated bacteria over 72 hours. 0.1 OD = $2.4x10^6$ bacterial cells/ml. *t*-test is a comparison with control. (* $p< 0.005$) (b) Area of 3D spheroids after incubation with 0.4 OD and 0.8 OD of inactivated bacteria, at the 0, 24, 48 and 72 hour time points. The area (μm^2) of the spheroids in the images was measured using ImageJ software. *t*-test is a comparison with control (* $p< 0.005$, at all time points). (c) Effect of 0.4 OD and 0.8 OD of inactivated *C. sporogenes* on 3D spheroids, compared with Control. Scale bar represents 1000 µm.
